# Prediction models of colorectal cancer prognosis incorporating perioperative longitudinal serum tumor markers: a retrospective longitudinal cohort study

**DOI:** 10.1186/s12916-023-02773-2

**Published:** 2023-02-21

**Authors:** Chunxia Li, Ke Zhao, Dafu Zhang, Xiaolin Pang, Hongjiang Pu, Ming Lei, Bingbing Fan, Jiali Lv, Dingyun You, Zhenhui Li, Tao Zhang

**Affiliations:** 1 Department of Biostatistics, School of Public Health, Cheeloo College of Medicine, Shandong University, 44 Wenhuaxi Road, PO Box 100, Jinan, 250012 Shandong China; 2 Department of Radiology, Guangdong Provincial People’s Hospital (Guangdong Academy of Medical Sciences), Southern Medical University, Guangzhou, 510080 China; 3 Guangdong Cardiovascular Institute, Guangzhou, 510080 China; 4 Guangdong Provincial Key Laboratory of Artificial Intelligence in Medical Image Analysis and Application, Guangdong Provincial People’s Hospital, Guangdong Academy of Medical Sciences, Guangzhou, 510080 China; 5 Department of Radiology, the Third Affiliated Hospital of Kunming Medical University, Yunnan Cancer Hospital, Yunnan Cancer Center, No.519 Kunzhou Road, Xishan District, Kunming, 650118 Yunnan China; 6 Department of Radiotherapy, the Sixth Affiliated Hospital of Sun Yat-Sen University, Guangzhou, 510655 China; 7 Department of Colorectal Surgery, the Third Affiliated Hospital of Kunming Medical University, Yunnan Cancer Hospital, Yunnan Cancer Center, Kunming, 650118 China; 8 Department of Clinical Laboratory Medicine, the Third Affiliated Hospital of Kunming Medical University, Yunnan Cancer Hospital, Yunnan Cancer Center, Kunming, 650118 China; 9 School of Biomedical Engineering Research, Kunming Medical University, No.1168 Chunrongxi Road, Chenggong District, Kunming, 650500 Yunnan China; 10 Institute for Medical Dataology, Shandong University, Jinan, 250002 China

**Keywords:** Colorectal cancer, Perioperative serum tumor markers, Dynamic prediction, Overall survival

## Abstract

**Background:**

Current prognostic prediction models of colorectal cancer (CRC) include only the preoperative measurement of tumor markers, with their available repeated postoperative measurements underutilized. CRC prognostic prediction models were constructed in this study to clarify whether and to what extent the inclusion of perioperative longitudinal measurements of CEA, CA19-9, and CA125 can improve the model performance, and perform a dynamic prediction.

**Methods:**

The training and validating cohort included 1453 and 444 CRC patients who underwent curative resection, with preoperative measurement and two or more measurements within 12 months after surgery, respectively. Prediction models to predict CRC overall survival were constructed with demographic and clinicopathological variables, by incorporating preoperative CEA, CA19-9, and CA125, as well as their perioperative longitudinal measurements.

**Results:**

In internal validation, the model with preoperative CEA, CA19-9, and CA125 outperformed the model including CEA only, with the better area under the receiver operating characteristic curves (AUCs: 0.774 vs 0.716), brier scores (BSs: 0.057 vs 0.058), and net reclassification improvement (NRI = 33.5%, 95% CI: 12.3 ~ 54.8%) at 36 months after surgery. Furthermore, the prediction models, by incorporating longitudinal measurements of CEA, CA19-9, and CA125 within 12 months after surgery, had improved prediction accuracy, with higher AUC (0.849) and lower BS (0.049). Compared with preoperative models, the model incorporating longitudinal measurements of the three markers had significant NRI (40.8%, 95% CI: 19.6 to 62.1%) at 36 months after surgery. External validation showed similar results to internal validation. The proposed longitudinal prediction model can provide a personalized dynamic prediction for a new patient, with estimated survival probability updated when a new measurement is collected during 12 months after surgery.

**Conclusions:**

Prediction models including longitudinal measurements of CEA, CA19-9, and CA125 have improved accuracy in predicting the prognosis of CRC patients. We recommend repeated measurements of CEA, CA19-9, and CA125 in the surveillance of CRC prognosis.

**Supplementary Information:**

The online version contains supplementary material available at 10.1186/s12916-023-02773-2.

Colorectal cancer (CRC) is a common malignancy, with high morbidity and mortality [1]. Currently, early colorectal cancer is mainly treated by radical resection, and clinicians formulate postoperative monitoring strategies based on the American Joint Committee on Cancer (AJCC) staging [2, 3]. However, patients with the same AJCC stage were reported to have heterogeneous survival outcomes [4]. So, for a more precise identification of high-risk patients with poor prognoses, individualized prognostic prediction is needed [4, 5].

Recently, prediction models with serum tumor markers, along with demographic and clinicopathological variables, have been widely constructed for prognosis prediction of CRC. Carcinoembryonic antigen (CEA), a recognized tumor marker in CRC [6, 7], has been included in prognostic models [4, 5]. In addition to CEA, the carbohydrate antigen 19–9 (CA19-9) and carbohydrate antigen 125 (CA125) have been reported to be associated with the prognosis of CRC [8–11]. However, whether and to what extent the further inclusion of CA19-9 and CA125 can improve the performance of the prediction model with CEA has not yet been clarified.

Current prognostic prediction models of CRC include only the preoperative measurement of tumor markers [5, 12, 13], with available repeated postoperative measurements of these markers underutilized. Studies have shown that longitudinal changing patterns of markers were prognostic factors independent of preoperative levels [14–16]. Therefore, we suppose that including the longitudinal measurements of tumor markers may be able to improve the prediction accuracy of the prognostic model.

Most of the existing prognostic nomograms for CRC are based on static models developed with baseline information [5, 13]. Once the model is constructed, the predicted risk of an individual will remain constant. However, disease prognosis is a dynamic process. Dynamic predictions are necessary, with risk predictions updated immediately to reflect the patient’s latest prognosis whenever new measurements of markers become available.

In this paper, we constructed prediction models for overall survival by random survival forest (RSF) [17], a nonparametric method shown to be robust for covariates with nonlinear effects and complex interactions in modeling time-to-event data [18–20], to evaluate the value of CEA, CA19-9, and CA125 on CRC prognostic assessment more intuitively. To better guide the prognostic management of colorectal cancer, a novel longitudinal prediction model incorporating prognostic information provided by repeated marker measurements will be proposed, and a clinically applicable dynamic prediction tool allowing prognostic predictions to be updated with follow-up will be developed.

## Methods

### Patients

**Fig. 1:**
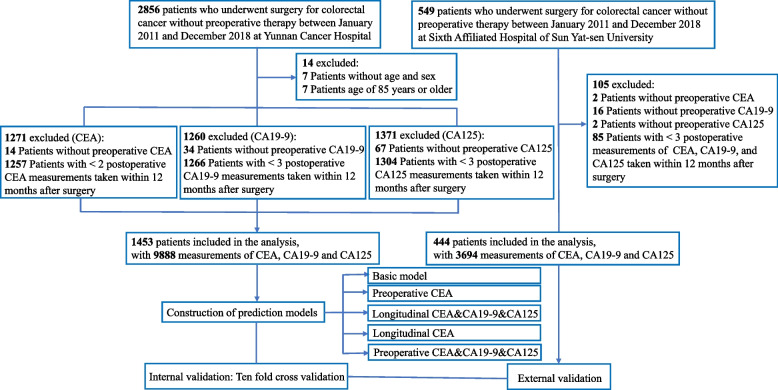
Study flowchart

All consecutive CRC patients without neoadjuvant treatment, undergoing curative resection for stage I to III colorectal adenocarcinoma between January 2011 and February 2019, were retrospectively identified from Yunnan Cancer Hospital (YNCH) and the Sixth Affiliated Hospital of Sun Yat-sen University (SYSU6). Patients with preoperative measurement and two or more measurements within 12 months after surgery were included in the construction of the prediction model. The inclusion and exclusion processes are shown in Fig. 1. Baseline characteristics of patients included and excluded are shown in Additional file 1. Data from YNCH were used for the construction of prediction models and internal validation, and data from SYSU6 were used for external validation.

### Serum marker determination

Preoperative measurement was defined as the value closest to the time of surgery within 4 weeks before surgery, and postoperative measurements were repeatedly measured after surgery, with different intervals and times for individuals. All measurements were made by chemiluminescence immunoassay using the COBAS 800 e602 immunoassay analyzer (Roche Diagnostics, Tokyo, Japan) at YNCH, and Alinity immunoassay analyzer (Abbott Diagnostics, Chicago, USA) at SYSU6, following World Health Organization standard methods (code 73/601).

### Covariates

Time-independent variables included preoperative CEA, preoperative CA19-9, preoperative CA125, age, sex, surgical approach (open resection or laparoscopic resection), primary site (colon or rectum), tumor differentiation, TNM stage, lymph node yield, mucinous (colloid) type, lymphovascular invasion, perineural invasion, and adjuvant chemotherapy. Time-dependent variables included longitudinal measurements of CEA, CA19-9, and CA125.

### Outcomes

In this retrospective study, the follow-up ended on June 30, 2020. The endpoint was overall survival (OS). The overall survival time was calculated from the date of surgery to the date of death or the last follow-up. Patients who were alive until the last follow-up were censored.

### Statistical analysis

Continuous variables were described as median [quartile], and categorical variables were described as number (percentage). Characteristics across patients in YNCH and SYSU6 were compared using the Mann–Whitney *U* test or Student *T*-test according to the normality assumption for continuous variables, and *χ*^2^ test for categorical variables. Survival curves for preoperative and the first postoperative CEA, CA19-9, and CA125 groups were drawn using the OS estimated by Kaplan–Meier, and log-rank test was performed to determine the overall difference between groups.

### Extraction of trajectory features

Due to the high intra-variability of these tumor markers, we set a maximum value for CEA, CA19-9, and CA125 to facilitate the extraction of trajectory features, which is ten times the upper limit of the reference range [21]. The log-transformed measurements were used as the longitudinal outcomes.

A functional principal component analysis (FPCA) [22, 23] is used to extract trajectory patterns of perioperative CEA, CA19-9, and CA125. FPCA smooths the sparse longitudinal measurements with irregular time intervals as functional curves, decomposed as a mean function and a summation of products of FPC scores and the corresponding eigenfunctions by Karhunen–Loeve expansion. And the calculated individual-specific functional principal component (FPC) scores can reflect different trajectory patterns. The optimal number of FPCs is determined based on the AIC criterion. Let ${Y}_{ij}$ and ${X}_{ij}$ be the observed and true longitudinal marker measurements at time ${t}_{ij}$. The FPCA can be described as:

$${Y}_{ij}={X}_{i}\left({t}_{ij}\right)+{\in }_{ij}=\mu \left({t}_{ij}\right)+\sum_{k=1}^{K}{\xi }_{ik}{\widehat{\phi }}_{k}\left({t}_{ij}\right)+{\epsilon }_{ij}$$

where $\mu \left({t}_{ij}\right)$ is the smoothed mean function, $\left\{{\left.{\xi }_{ik}\right\}}_{k=1,\dots ,K}\right.$ are the FPC scores, $\left\{{\phi }_{k}\right.{\left.\left({t}_{ij}\right)\right\}}_{l=1,\dots ,K}$ are the corresponding orthonormal eigenfunctions, ${\in }_{ij}$ is a measurement error term, and *K* is the number of FPCs. *Y *can be longitudinal measurements of CEA, CA19-9, or CA125.

In consideration of the correlation between the FPC scores of CEA, CA19-9, and CA125, multivariate principal components analysis (MFPCA) [24, 25] was applied to characterize the changing patterns of the multivariate longitudinal processes. MFPCA indirectly modeled the correlations among the three tumor markers via the correlations among their FPC scores.

### Construction of prediction models

Random Survival Forest (RSF) is an extension of the random forest method for right-censored survival data, constructed by aggregating an ensemble of survival trees [17, 18, 26]. Compared with traditional survival methods, the modeling of RSF is more flexible, requiring no restrictive assumptions. In consideration of longitudinal markers with nonlinear trajectories and possible interactions among multiple covariates, RSF without the need for prior specification is a good choice to develop prediction models in this study.

“randomForestSRC” is a comprehensive R package developed to compute random forests for survival, regression, and classification, providing a fast OpenMP parallel computing to improve modeling efficiency. RSF modeling is easily implementable using the rfsrc function, which is the main entry point to the “randomForestSRC” package, and users can read the help file in its entirety for details.

The random survival forest model was constructed for survival prediction. FPC scores as well as demographic and clinicopathological variables were all candidate variables. Variable importance (VIMP) was calculated to rank variables based on predictive ability. Five prediction models were developed. The basic model included demographic variables (age and sex) and clinicopathological variables (primary site, surgical approach, tumor differentiation, histological type, pathology stage, lymph node yield, adjuvant chemotherapy, mucinous (colloid) type, lymphovascular invasion, and perineural invasion) for prediction. For the preoperative CEA and preoperative CEA&CA19-9&CA125 model, preoperative levels of CEA and all three tumor markers were incorporated into the basic model. For longitudinal CEA and longitudinal CEA&CA19-9&CA125 model, preoperative levels of CEA and all three tumor markers, as well as their longitudinal trajectory features within 12 months after surgery, were incorporated into the basic model, respectively. In addition, a postoperative CEA&CA19-9&CA125 model was also constructed, with the first postoperative measurements of CEA, CA19-9, and CA125 incorporated into the preoperative CEA&CA19-9&CA125 model.

### Evaluation of prediction models

For internal validation, prediction models were validated with tenfold cross-validation, based on data from Yunnan Cancer Hospital. The landmark time was specified as 12 months after surgery. Survival probability was estimated from 18 to 60 months after surgery. Both discrimination and calibration were assessed to evaluate the accuracy of the prediction models. Discrimination was measured by area under the receiver operating characteristic curve (AUC) over time, and calibration was measured by brier score (BS). Higher AUC and lower BS indicate better prediction performance [27]. The net reclassification improvement (NRI) [28] and integrated discrimination improvement (IDI) [29] were estimated to quantify how well a new model reclassifies individuals in terms of predicted risk.

External validation was also performed, based on data from the Sixth Affiliated Hospital of Sun Yat-sen University. The landmark time was specified as 12 months after surgery. Survival probability was estimated at 60 months after surgery. Predictive validity was assessed using receiver operating characteristic (ROC) curves.

### Dynamic prediction

For a target patient, whenever a new marker measurement is obtained, the functional principal component scores are re-estimated, so the predicted survival probability can be updated. Let the observed event time ${T}_{N}^{*}=min\left\{{T}_{N},\left.{C}_{N}\right\}\right.$, where ${T}_{N}$ is the true event time, and ${C}_{N}$ is the censoring time independent from ${T}_{N}$. The survival probability can be expressed as a conditional probability:

$$\hat{\pi }_{N} \left( {s^{\prime } |s} \right) = p\left( {T_{N}^{*} \ge s^{\prime } |T_{N}^{*} > s,Z_{N} ,\widehat{\xi }_{N} } \right)$$

where ${T}^{*}$ is the observed event time, ${Z}_{N}$ aree the time-independent covariates, and ${\widehat{\xi }}_{N}$ are the MFPCA scores.

To illustrate the personalized dynamic predictions, we set aside three target patients (Patient A, B, and C) with CRC of stages I–III and predict their CEA levels and future survival probability using the CEA&CA19-9&CA125 model. The prediction model used for each target patient was trained using the data that remained in the YNCH after excluding this target patient. And an interactive web application was constructed to implement dynamic prediction of a new CRC patient, based on the longitudinal CEA&CA19-9&CA125 model.

All statistical analyses mentioned above were performed using R software (version 3.6.3; http://www.R-project.org). FPCA was implemented with package “fdapace” (Version 0.5.5), MFPCA was implemented with package “MFPCA” (Version 1.3–6), and the RSF model was constructed with package “randomForestSRC” (Version 2.10.1).

## Results

### Patient characteristics

Of the 2856 patients who underwent surgery for colorectal cancer without preoperative therapy between January 2011 and December 2018 at Yunnan Cancer Hospital, 1453 patients were included, with 9888 measurements of tumor markers. Substantially, the clinical characteristics of patients included and excluded were similar (Additional file 1). The YNCH cohort of 1453 patients included 861 (59.3%) men, with a median (interquartile range [IQR]) age of 58.0 [49.0, 65.0] years (Table 1) and a median follow-up time of 44.7 months ([IQR]: 28.8–63.7 months). During follow-up, a total of 176 patients (12.1%) died, with an incidence density of 87.77 per 1000 person-years.

**Table 1:** Characteristics by the two cohorts of CRC patients

Variable	YNCH (*n* = 1453)	SYSU6 (*n* = 444)	*P* value
**Preoperative**			
Preoperative CEA	3.9 [2.2, 9.0]	3.5 [1.9, 9.5]	0.120
Preoperative CA19-9	12.6 [7.5, 22.9]	13.0 [6.1, 34.5]	0.054
Preoperative CA125	12.9 [9.0, 18.7]	11.5 [7.9, 17.4]	0.002
**Postoperative**			
Postoperative CEA	1.9 [1.2, 2.9]	1.9 [1.2, 2.8]	0.807
Postoperative CA19-9	9.4 [5.9, 15.9]	7.2 [4.0, 13.5]	< 0.001
Postoperative CA125	24.5 [16.2, 39.3]	18.1 [11.0, 31.4]	< 0.001
**Covariate**			
Age	58.0 [49.0, 65.0]	57.0 [47.0, 63.0]	0.055
Male, *n* (%)	861 (59.3)	259 (58.3)	0.771
Primary site			< 0.001
Colon, *n* (%)	746 (51.3)	341 (76.8)	
Rectum,* n* (%)	707 (48.7)	103 (23.2)	
Surgical approach			< 0.001
Laparoscopic resection, *n* (%)	551 (37.9)	381 (85.8)	
Open resection, *n* (%)	902 (62.1)	63 (14.2)	
Tumor differentiation			< 0.001
Well, *n* (%)	8 (0.6)	74 (16.7)	
Moderate, *n* (%)	908 (62.5)	246 (55.4)	
Poor-undifferentiated, *n* (%)	454 (31.2)	124 (27.9)	
Unknown, *n* (%)	83 (5.7)	0 (0.0)	
AJCC 8th ed. Stage			0.195
I, *n* (%)	189 (13.0)	71 (16.0)	
II,* n* (%)	584 (40.2)	163 (36.7)	
III, *n* (%)	680 (46.8)	210 (47.3)	
Lymph node yield			< 0.001
< 12, *n* (%)	287 (19.8)	36 (8.1)	
≥ 12, *n* (%)	1166 (80.2)	408 (91.9)	
Mucinous (colloid) type	98 (6.7)	29 (6.5)	0.961
Lymphovascular invasion	123 (8.5)	56 (12.6)	0.012
Perineural invasion	30 (2.1)	83 (18.7)	< 0.001
Adjuvant chemotherapy	1252 (86.2)	415 (93.5)	< 0.001

Data are median [IQR], or *n* (%)

*YNCH* Yunnan Cancer Hospital, *SYSU6* the Sixth Affiliated Hospital of Sun Yat-sen University

Of the 549 patients who underwent surgery for colorectal cancer without preoperative therapy between January 2011 and December 2018 at the Sixth Affiliated Hospital of Sun Yat-sen University, 444 patients were included, with 3694 measurements of tumor markers. The distribution of baseline characteristics of patients included was substantially consistent with that of those excluded (Additional file 1). The SYSU6 cohort of 444 patients included 259 (58.3%) men, with a median (interquartile range [IQR]) age of 57.0 [47.0, 63.0] years (Table 1) and a median follow-up time of 40.6 months ([IQR]: 37.1–48.9 months). During follow-up, a total of 33 patients (7.4%) died, with an incidence density of 49.99 per 1000 person-years.

The characteristics of patients by the two cohorts are outlined in Table 1. Compared with patients in the YNCH, patients in the SYSU6 had higher proportions of colon cancer, laparoscopic resection, well tumor differentiation, ≥ 12 lymph node yield, lymphovascular invasion, perineural invasion, and adjuvant chemotherapy. In terms of age, gender, pathological stage, and mucinous (colloid) type, the two cohorts did not show significant differences (Table 1).

Overall, the first postoperative levels of CEA and CA19-9 were lower than their preoperative levels, while the first postoperative CA125 was higher than preoperative CA125 (Table 1). And patients with elevated preoperative or postoperative CEA (> 5 ng/ml), CA19-9 (> 37 U/ml), and CA125(> 35 U/ml) had poorer overall survival compared to those with normal preoperative or postoperative CEA, CA19-9, and CA125 (Additional File 2: Figures S1-S2).

### Extraction of longitudinal features

The smoothed curves of longitudinal CEA, CA19-9, and CA125 of survived and dead patients are shown in Additional File 2: Figure S3. People who died tended to have higher postoperative CEA, CA19-9, and CA125 within 12 months after surgery. In the extraction of trajectory features, the first 6, 6, and 5 principal components for longitudinal CEA, CA19-9, and CA125 were selected based on Akaike Information Criterion (AIC), respectively. Corresponding eigenfunctions are shown in Additional File 2: Figure S4. And the first 17 principal components of the three longitudinal markers were selected in MFPCA, with the first five eigenfunctions shown in Additional File 2: Figure S5. Additional File 2: Figure S6 displays the relative importance of the first 10 important variables ranked by VIMP in the preoperative CEA&CA19-9&CA125 model and longitudinal CEA&CA19-9&CA125 model. The first and second principal components of the three tumor markers’ perioperative measurements were important prognostic predictors, second only to the pathological stage.

### Internal verification of prediction models

**Fig. 2:**
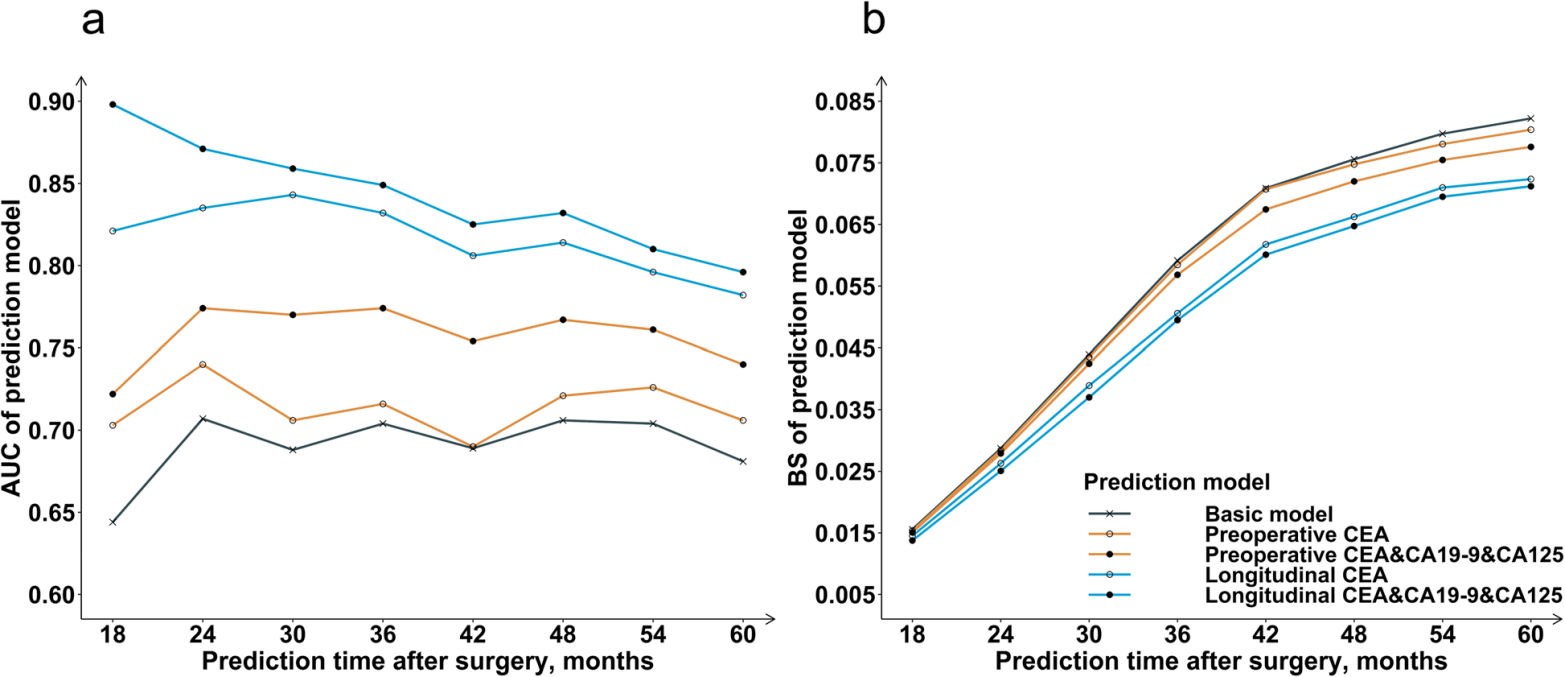
AUC (**a**) and BS (**b**) of the prediction models at 18 to 60 months after surgery for internal validation. AUC, area under the receiver operating characteristic curve; BS, brier score

The AUCs for the basic model were 0.707 at 24 months, 0.704 at 36 months, 0.706 at 48 months, and 0.681 at 60 months. The BSs for the basic model were 0.029 at 24 months, 0.059 at 36 months, 0.076 at 48 months, and 0.082 at 60 months (Fig. 2).

The prediction performance of the preoperative CEA model was enhanced with the inclusion of preoperative CA19-9 and CA125. At each prediction time (18 to 60 months after surgery), the preoperative CEA&CA19-9&CA125 model had higher AUC and lower BS than the preoperative CEA model (Fig. 2). Specifically, the AUCs for the preoperative CEA and preoperative CEA&CA19-9&CA125 model were 0.740 vs 0.774 at 24 months, 0.716 vs 0.774 at 36 months, 0.721 vs 0.767 at 48 months, and 0.706 vs 0.740 at 60 months. And the BSs for the two preoperative models were 0.028 vs 0.028 at 24 months, 0.058 vs 0.057 at 36 months, 0.075 vs 0.072 at 48 months, and 0.080 vs 0.078 at 60 months.

Compared with the preoperative CEA model, the preoperative CEA-CA19-9-CA125 model had improved accuracy in risk estimates. The NRI (95% CI) were 34.05% (3.29%, 64.81%) at 24 months, 33.53% (12.28%, 54.78%) at 36 months, 36.04% (17.18%, 54.90%) at 48 months, and 21.47% (2.95%, 39.99%) at 60 months (Table 2). And the corresponding IDI (95% CI) were 0.011 (0.001, 0.021), 0.017 (0.005, 0.029), 0.024 (0.009, 0.039), and 0.020 (0.006, 0.035) (Table 3).

**Table 2:** Net reclassification improvement of prediction models

**Preoperative CEA&CA19-9&CA125 model vs preoperative CEA model**				
	Percentage of individuals for whom the preoperative CEA&CA19-9&CA125 model estimates a higher risk than the preoperative CEA model	Percentage of individuals for whom the preoperative CEA&CA19-9&CA125 model estimates a lower risk than the preoperative CEA model	Overall net reclassification improvement (95% confidence interval)	*P* for net reclassification improvement
24 months				
Events	64.29%	35.71%	34.05% (3.29%, 64.81%)	0.015
Non-events	47.26%	52.74%		
36 months				
Events	59.57%	40.43%	33.53% (12.28%, 54.78%)	0.001
Non-events	42.81%	57.19%		
48 months				
Events	60.47%	39.53%	36.04% (17.18%, 54.90%)	< 0.001
Non-events	42.45%	57.55%		
60 months				
Events	57.62%	42.38%	21.47% (2.95%, 39.99%)	0.012
Non-events	46.88%	53.12%		
**Longitudinal CEA&CA19-9&CA125 model vs preoperative CEA&CA19-9&CA125 model**				
	Percentage of individuals for whom the longitudinal CEA&CA19-9&CA125 model estimates a higher risk than the preoperative CEA&CA19-9&CA125 model	Percentage of individuals for whom the longitudinal CEA&CA19-9&CA125 model estimates a lower risk than the preoperative CEA&CA19-9&CA125 model	Overall net reclassification improvement (95% confidence interval)	*P* for net reclassification improvement
24 months				
Events	73.81%	26.19%	64.87% (34.11%, 95.63%)	< 0.001
Non-events	41.37%	58.63%		
36 months				
Events	62.77%	37.23%	40.81% (19.56%, 62.06%)	< 0.001
Non-events	42.36%	57.64%		
48 months				
Events	60.47%	39.53%	30.30% (11.44%, 49.16%)	0.001
Non-events	45.32%	54.68%		
60 months				
Events	56.95%	43.05%	24.76% (6.24%, 43.28%)	0.004
Non-events	44.57%	55.43%		

Preoperative CEA model included age and sex, primary site, surgical approach, tumor differentiation, histological type, pathology stage, lymph node yield, adjuvant chemotherapy, mucinous (colloid) type, lymphovascular invasion and perineural invasion, and preoperative CEA for prediction. Preoperative CEA&CA19-9&CA125 model included age and sex, primary site, surgical approach, tumor differentiation, histological type, pathology stage, lymph node yield, adjuvant chemotherapy, mucinous (colloid) type, lymphovascular invasion and perineural invasion, and preoperative CEA for prediction. Longitudinal CEA&CA19-9&CA125 model further included preoperative CA19-9 and CA125 in addition to variables in preoperative CEA&CA19-9&CA125 model

**Table 3:** Integrated discrimination improvement of prediction models

**Preoperative CEA&CA19-9&CA125 model vs preoperative CEA model**				
	Mean predicted probability of preoperative CEA&CA19-9&CA125 model	Mean predicted probability of preoperative CEA model	Overall integrated discrimination improvement (95% confidence interval)	*P* for integrated discrimination improvement
24 months				
Events	0.072	0.061	0.011 (0.001, 0.021)	0.016
Non-events	0.031	0.031		
36 months				
Events	0.159	0.143	0.017 (0.005, 0.029)	0.003
Non-events	0.074	0.074		
48 months				
Events	0.219	0.197	0.024 (0.009, 0.038)	0.001
Non-events	0.109	0.111		
60 months				
Events	0.257	0.237	0.020 (0.006, 0.035)	0.004
Non-events	0.142	0.143		
**Longitudinal CEA&CA19-9&CA125 model vs preoperative CEA&CA19-9&CA125 model**				
	Mean predicted probability of longitudinal CEA&CA19-9&CA125 model	Mean predicted probability of preoperative CEA&CA19-9&CA125 model	Overall integrated discrimination improvement (95% confidence interval)	*P* for integrated discrimination improvement
24 months				
Events	0.132	0.072	0.062 (0.038, 0.085)	< 0.001
Non-events	0.029	0.031		
36 months				
Events	0.226	0.159	0.071 (0.045, 0.098)	< 0.001
Non-events	0.069	0.074		
48 months				
Events	0.267	0.219	0.053 (0.030, 0.076)	< 0.001
Non-events	0.105	0.109		
60 months				
Events	0.292	0.257	0.040 (0.018, 0.061)	< 0.001
Non-events	0.137	0.142		

Preoperative CEA model included age and sex, primary site, surgical approach, tumor differentiation, histological type, pathology stage, lymph node yield, adjuvant chemotherapy, mucinous (colloid) type, lymphovascular invasion and perineural invasion, and preoperative CEA for prediction. Preoperative CEA&CA19-9&CA125 model included age and sex, primary site, surgical approach, tumor differentiation, histological type, pathology stage, lymph node yield, adjuvant chemotherapy, mucinous (colloid) type, lymphovascular invasion and perineural invasion, and preoperative CEA for prediction. Longitudinal CEA&CA19-9&CA125 model further included preoperative CA19-9 and CA125 in addition to variables in preoperative CEA&CA19-9&CA125 model

Similar to preoperative models, the prediction performance of the longitudinal CEA model was enhanced with the inclusion of longitudinal measurements of CA19-9 and CA125. Both discrimination and calibration of the longitudinal CEA&CA19-9&CA125 model were better than that of the longitudinal CEA model (Fig. 2). The AUCs for the longitudinal CEA and longitudinal CEA&CA19-9&CA125 model were 0.835 vs 0.871 at 24 months, 0.832 to 0.849 vs 36 months, 0.814 vs 0.832 at 48 months, and 0.782 vs 0.796 at 60 months. And the BSs for the three longitudinal models decreased from 0.026 to 0.025 at 24 months, from 0.051 to 0.049 at 36 months, from 0.066 to 0.065 at 48 months, and from 0.072 to 0.071 at 60 months.

Compared with the preoperative model, the prediction accuracy of the longitudinal model incorporating the repeated measurements of CEA, CA19-9, or CA125 was improved. The longitudinal CEA model performed better than the preoperative CEA model in both AUC and BS (Fig. 2). With the further inclusion of longitudinal CEA, the AUC for the preoperative CEA model increased from 0.740 to 0.835 at 24 months, from 0.716 to 0.832 at 36 months, from 0.721 to 0.814 at 48 months, and from 0.706 to 0.782 at 60 months. And the BS decreased from 0.028 to 0.026 at 24 months, from 0.058 to 0.051 at 36 months, from 0.075 to 0.066 at 48 months, and from 0.080 to 0.072 at 60 months. Likewise, the longitudinal CEA&CA19-9&CA125 model performed better than the preoperative CEA&CA19-9&CA125 model in predicting the overall survival of CRC patients.

Compared with the preoperative CEA&CA19-9&CA125 model, the longitudinal CEA&CA19-9&CA125 model had improved accuracy in risk estimates. The NRI (95% CI) were 64.87% (34.11%, 95.63%) at 24 months, 40.81% (19.56%, 62.06%) at 36 months, 30.30% (11.44%, 49.16%) at 48 months, and 24.76% (6.24%, 43.28%) at 60 months (Table 2). And the corresponding IDI (95% CI) were 0.062 (0.038, 0.085), 0.071 (0.045, 0.098), 0.053 (0.030, 0.076), and 0.040 (0.019, 0.061) (Table 3). In addition, the AUC for the postoperative CEA&CA19-9&CA125 model was 0.762 at 60 months after surgery, which was higher than the preoperative CEA&CA19-9&CA125 model (AUC = 0.740) and lower than the longitudinal CEA&CA19-9&CA125 model (AUC = 0.796) (Additional File 2: Figure S7a).

### External verification of prediction models

**Fig. 3:**
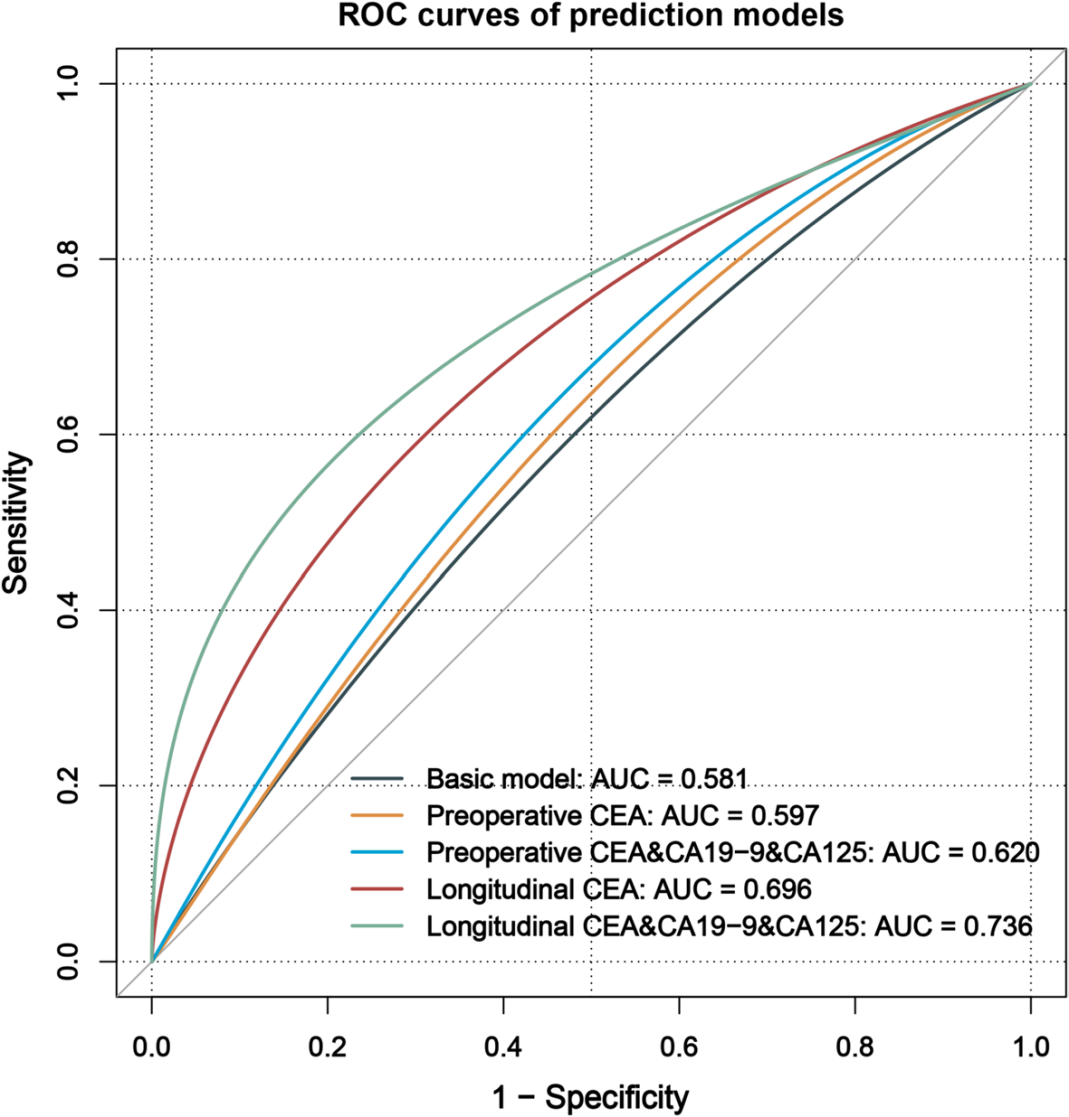
ROC curves of the prediction models at 60 months after surgery for external validation. AUC, area under the receiver operating characteristic curve

External validation showed similar results to the above internal validation. The ROC curves of the five prediction models, using the YNCH cohort for training and the SYSU6 cohort for validation, are shown in Fig. 3. At 60 months after surgery, the AUCs for the basic model, preoperative CEA model, preoperative CEA&CA19-9&CA125 model, longitudinal CEA model, and longitudinal CEA&CA19-9&CA125 model were 0.581, 0.597, 0.620, 0.696, and 0.736. Although the discriminative accuracy of these prediction models reduced in external verification, the AUC improved with the incorporation of tumor markers and their longitudinal information. And the performance of the preoperative, postoperative, and longitudinal CEA&CA19-9&CA125 model was also gradually improved in external validation, with the AUCs to be 0.620, 0.638, 0.736 (Additional File 2: Figure S7b).

### Personalized dynamic prediction

Patient A, with colon cancer of stage I, survived 71.6 months after surgery. Patient B, with colon cancer of stage II, died 42.57 months after surgery. Patient C, with poorly differentiated rectal cancer of stage III, died at 15.3 months after surgery. The personalized dynamic predictions of patient A, patient B, and patient C are displayed in Additional File 2: Figure S8. The CEA level estimated by the longitudinal CEA&CA19-9&CA125 model was close to the observed measurement, and the predicted risk was closer to the real situation with the gradual inclusion of tumor measurements. Concretely, the postoperative CEA of patient A decreased and remained at a low level, with a flatter rate of decrease of the conditional survival function, while the postoperative CEA of patient C gradually increased, with a steeper rate of decrease of the conditional survival function. The CEA of Patient B decreased firstly and then increased, with the conditional survival function decreasing slowly and then rapidly. And the conditional survival function curve of Patient B was lower than that of patient A and higher than that of patient C. The shiny app of the proposed model has been submitted to github [30], and the command “shiny::runGitHub (“Dynamic-prediction-of-overall-survival”, username = “ccckyx”, ref = “main”)” will run it in R.

## Discussion

In this study, we constructed prediction models to evaluate the prognostic value of serum tumor markers in a more intuitive way and found that the predictive performance of CRC prognostic models improved with the incorporation of preoperative CEA, CA19-9, and CA125. The functional data analysis method was innovatively applied to sparse and irregular repeated marker measurements to extract individual-specific longitudinal features, and longitudinal models were created, which were proven to have better discrimination and calibration than preoperative models. The proposed longitudinal prediction model based on MFPCA can provide a personalized dynamic prediction for a new patient, with estimated survival probability updated when a new measurement is collected during 12 months after surgery. The dynamic prediction tool developed based on the model had high clinical applicability, enabling the repeated measurement of perioperative CEA, CA19-9, and CA125 to be fully utilized, which will benefit the prognosis assessment and treatment management of CRC patients.

The improved prediction accuracy of preoperative models with the inclusion of multiple prognostic tumor markers was supported by previous studies. It has been reported that CRC patients with simultaneously positive preoperative CEA, CA19-9, and CA125 tended to have the highest rate and the shortest survival time for death [31] and recurrence [32]. The number of elevated preoperative tumor markers was concluded to significantly predict the prognosis of patients with stage II and III CRC [33]. Recently, attention has been paid to prediction models incorporating multiple tumor markers. Tang et al. developed a predictive nomogram to identify early recurrence, with CA19-9 and CA125 included. Zhu et al. confirmed the clinical implications of CEA, CA19-9, CA125, and positive lymph node scheme (LODDS) in predicting OS of CRC patients and conducted a novel nomogram incorporating the three tumor markers and LODDS. This novel proposed model performed better than the model containing CEA only, consistent with our results [13]. However, the extent to which the further addition of CA19-9 and CA125 to the model including CEA can improve the prediction has not been quantified. In our study, based on the random survival forest model, two preoperative models including different numbers of tumor markers were constructed. Compared with the model with only preoperative CEA, the model including all three tumor markers has significantly improved predictive accuracy. Our results supplement previous research, making the prediction improvement of the combination of markers more intuitive.

In addition to the preoperative level, the dynamic measurements of tumor markers during follow-up after surgery also provide important prognostic information. The changing patterns of postoperative tumor markers are important prognostic predictors [14]. Patients whose CEA continues to rise after surgery have a higher risk of death or recurrence than those whose CEA declines and remains stable [15, 16]. Ma et al. studied the impact of dynamic changes in inflammation and biochemical indicators on the prognosis of perioperative patients and constructed a new prognostic model using dynamic changes to achieve a more accurate prediction of the overall survival and disease-free survival of patients with CRC [34]. Though this predictive model contains dynamic information, limited measurements of indicators were used. And few previous studies incorporated dynamic measurements of tumor markers into prognostic models of CRC. In our prediction model, the longitudinal changing features of perioperative CEA, CA19-9, and CA125 within 12 months after surgery were expressed as principal component scores and included in the survival model for dynamic prediction. Compared with the model including preoperative levels, the predictive performance of the longitudinal model has been greatly improved. Through the comparison between the preoperative CEA&CA19-9&CA125 model and the longitudinal CEA&CA19-9&CA125 model, the prognostic value of the longitudinal changing patterns of the three markers was quantified.

For a specific individual, we expect to update the estimate of prognostic risk based on a newly obtained measurement of tumor marker, for which a dynamic prediction model is needed. It is worth noting that although the longitudinal prediction model incorporating repeated measurements of CEA, CA19-9, and CA125 within 12 months after surgery is not dynamic, it can be used for dynamic prediction. That is, dynamic prediction can be achieved by MFPCA-RSF. In the field of dynamic prediction, the joint model is a commonly used model that can process longitudinal data and survival data simultaneously [35]. Based on the joint model, Cao et al. have established a model to dynamically predict OS of patients with ovarian cancer, with longitudinal CA125 values considered [36]. However, the joint model fits longitudinal data using parametric models, whose parameter distribution is difficult to determine for longitudinal markers with complex patterns. Moreover, the traditional joint model cannot handle multiple longitudinal data. This limits the application of the joint model in our data. The functional data analysis method used in this study is non-parametric, making no assumption about the trajectory and being flexible in non-linear situations. It has been widely used in the feature extraction of longitudinal data. Yan et al. applied FPCA to longitudinal measurements of BCR-ABL gene expression levels to extract features, and use these features as covariates in a Cox proportional hazard model to conduct dynamic predictions of chronic myeloid leukemia [23]. And Luo et al. have extended FPCA to multivariate principal components analysis (MFPCA) and implemented dynamic predictions for Alzheimer’s disease and Parkinson’s disease [24, 25, 37, 38]. On the other hand, the joint model predicts future survival probability based on the Cox proportional hazard regression model, requiring the data to satisfy the proportional hazard assumption. The estimation of Cox model is unstable when there are complex interactions between variables. Therefore, we chose the RSF model, proven to remain robust in the presence of interactions between covariates [20]. However, FPCA-RSF is poor in interpretability. The meaning of the eigenfunctions and corresponding FPCs estimated by FPCA and MFPCA is difficult to explain, and the effect of the covariates on the survival outcome cannot be quantified in RSF [16]. Although we measured variable importance and found that the principal components were important predictors, the contribution of CEA, CA19-9, and CA125 to each principal component was not clear. MFPCA only focused on the common components of CEA, CA19-9, and CA125, with marker-specific components ignored [25].

Our study had several strengths. The performance improvement, brought by the further inclusion of CA19-9 and CA125 in the prognosis prediction model with CEA only, quantified the clinical value of simultaneous measurements of CEA, CA19-9, and CA125. The FPCA and MFPCA methods used to extract longitudinal features in our study are flexible, suitable for various and nonlinear changing patterns of tumor markers. Different from the trajectory analysis methods, the functional data analysis method can estimate longitudinal trajectory features which are individual-specific and time-varying. By extracting individual-specific longitudinal features, longitudinal prediction models containing more prognostic information were proposed, which allows clinicians to make full use of available repeated measurements of tumor markers to make more accurate prognostic predictions. Based on the time dependence of the extracted features, the longitudinal model can be used for dynamic prediction. For a target patient, the predicted risk can be updated timely to reflect the latent prognosis. In addition to time-dependent variables, time-varying effects of time-independent prognostic factors may also contribute to the changed prognosis [21]. And demographic and clinicopathological covariates with possible time-dependent effects have been considered in our study, using the random survival forest method which was robust when the proportional hazards assumption was violated [19].

Our study is subject to the limitations and bias inherent in observational retrospective studies. The inclusion criteria were patients who had at least three marker measurements within 12 months after surgery, which may lead to selection bias. Besides, the proposed dynamic prediction model was constructed and validated using data from South China only, and whether the model can be applied to a wider population still needs to be further verified. A large-scale, multicenter prospective cohort study with regular follow-up will provide more evidences to validate the model for dynamic prediction and increase the impact. In external verification, limited by the number of CRC patients in the SYSU6 cohort, we only predicted the survival probability at 60 months after surgery, to ensure a sufficient number of outcomes. The CRC patients in SYSU6 had a low mortality rate, related to the high economic and medical level of Guangdong province. Despite the differences between patients in YNCH and SYSU6, our proposed dynamic prediction model constructed using data from YNCH performed well for patients in SYSU6, indicating that the model had certain stability and extrapolation. MSI status is a well-known factor associated with the prognosis of colorectal cancer [39]. However, since MSI status was not widely detected until 2017 in Yunnan province with backward economic conditions, it had a high missing rate in the current study, and it was not considered when constructing our models. An exploratory analysis based on available MSI data showed that the predictive performance of prediction models including MSI status had a trend of improvement with the incorporation of CEA, CA19-9, and CA125 (Additional File 2: Figure S9). However, the value of including CEA, CA19-9, and CA125 in prediction models with MSI status needs to be further clarified in a large cohort. It should be mentioned that the proposed model is not suitable for patients with stage IV colorectal cancer. Patients with stage I–III and IV CRC receive different treatment regimens, with stage I–III patients mainly treated with surgical resection and stage IV patients mainly treated with chemotherapy and targeted therapy, resulting in heterogeneity in their changing patterns of tumor markers. Therefore, to reduce the complexity of prediction models, stage IV patients were not included in this study. However, the development of dynamic prediction models for stage IV CRC patients is also valuable and should be studied specifically in the future.

## Conclusions

In conclusion, the predictive performance of CRC prognostic models improved with the incorporation of preoperative CEA, CA19-9, and CA125, as well as their perioperative longitudinal measurements. The longitudinal measurements of CEA, CA19-9, and CA125 are recommended to predict the prognosis of CRC patients.

## Supplementary Information

**Additional file 1.** Characteristics of the included and excluded patients in YNCH and SYSU6.

**Additional file 2:** **Figure S1.** [Kaplan-Meier curves of overall survival for the preoperative groups of CEA  (a), CA19-9 (b), and CA125 (c) in colorectal cancer patients]. **Figure S2.** [Kaplan-Meier curves of overall survival for the first postoperative groups of CEA (a), CA1 -9 (b), and CA125 (c) in colorectal cancer patients]. **Figure S3.** [Longitudinal trajectories of CEA (a), CA19-9 (b) and CA125 (c) of survived and died patients]. **Figure S4.** [The eigenfunctions of FPCA for CEA (a), CA19-9 (b), and CA125 (c)]. **Figure S5.** [The first five eigenfunctions for CEA (a), CA19-9 (b), and CA125 (c) based on MFPCA of the three markers]. **Figure S6.** [Variable importance of preoperative CEA&CA19-9&CA125 model (a) and longitudinal CEA&CA19-9&CA125 model (b)]. **Figure S7.** [ROC curves of the preoperative, postoperative and longitudinal CEA&CA19-9&CA125 models at 60 months after surgery for internal validation (a) and external validation (b)]. **Figure S8.** [Personalized dynamic prediction for the survival probability of patient A (a), patient B (b) and patient C (c) based on longitudinal CEA&CA19-9&CA125 model]. **Figure S9.** [AUC (a) and BS (b) of the prediction models with MSI status at 24 to 60 months after surgery for external validation].

## Data Availability

The data underlying this article cannot be shared publicly due to individuals’ privacy that. participated in the study. The data will be shared on a reasonable request to the corresponding author.

## Abbreviations

AJCC: American Joint Committee on Cancer
AUC: Area under the receiver operating characteristic curve
BS: Brier score
CA125: Carbohydrate antigen 125
CA19-9: Carbohydrate antigen 19–9
CEA: Carcinoembryonic antigen
CRC: Colorectal cancer
FPC: Functional principal component
FPCA: Functional principal component analysis
IDI: Integrated discrimination improvement
MFPCA: Multivariate principal components analysis
NRI: Net reclassification improvement
OS: Overall survival
ROC curves: Receiver operating characteristic curves
RSF: Random survival forest
SYSU6: The Sixth Affiliated Hospital of Sun Yat-sen University
VIMP: Variable importance
YNCH: Yunnan Cancer Hospital
