# Supplementary material for: Prediction models of colorectal cancer prognosis incorporating perioperative longitudinal serum tumor markers: a retrospective longitudinal cohort study
Source: BMC Med. 2023 Feb 21;21:63. doi: 10.1186/s12916-023-02773-2 (PMC9942392; doi:10.1186/s12916-023-02773-2)
Supplement: Supplementary file 1 — Additional file 1. Characteristics of the included and excluded patients in YNCH and SYSU6. [file 12916_2023_2773_MOESM1_ESM.pdf]

Characteristics of the included and excluded patients in YNCH and SYSU6

Variable	YNCH		SYSU6	
	Included (n = 1453)	Excluded (n = 1403)	Included (n = 444)	Excluded (n = 105)
**Covariate**				
Preoperative CEA	3.9 [2.2, 9.0]	3.6 [2.1, 7.8]	3.5 [1.9, 9.5]	3.5 [1.9, 10.5]
Preoperative CA19-9	12.6 [7.5, 22.9]	13.2 [7.9, 23.1]	13.0 [6.1, 34.5]	3.1 [2.0, 9.2]
Preoperative CA125	12.9 [9.0, 18.7]	13.1 [9.6, 18.0]	11.5 [7.9, 17.4]	11.1 [7.8, 16.7]
Age	58.0 [49.0, 65.0]	63.0 [54.0, 70.0]	57.0 [47.0, 63.0]	57.0 [47.0, 64.0]
Male, n (%)	861 (59.3)	798 (57.2)	259 (58.3)	61 (58.1)
Primary site				
Colon, n (%)	746 (51.3)	636 (45.3)	341 (76.8)	52 (49.5)
Rectum, n (%)	707 (48.7)	767 (54.7)	103 (23.2)	53 (50.5)
Surgical approach				
Laparoscopic resection, n (%)	551 (37.9)	508 (36.2)	381 (85.8)	93 (88.6)
Open resection, n (%)	902 (62.1)	888 (63.3)	63 (14.2)	12 (11.4)
Unknown, n (%)	0 (0.0)	7 (0.5)	0 (0.0)	0 (0.0)
Tumor differentiation				
Well, n (%)	8 (0.6)	14 (1.0)	74 (16.7)	24 (22.9)
Moderate, n (%)	908 (62.5)	868 (61.9)	246 (55.4)	36 (34.3)
Poor-undifferentiated, n (%)	454 (31.2)	358 (25.5)	124 (27.9)	45 (42.9)
Unknown, n (%)	83 (5.7)	163 (11.6)	0 (0.0)	0 (0.0)
AJCC 8th ed. Stage				
I, n (%)	189 (13.0)	465 (33.1)	71 (16.0)	24 (22.9)
II, n (%)	584 (40.2)	546 (38.9)	163 (36.7)	41 (39.0)
III, n (%)	680 (46.8)	385 (27.4)	210 (47.3)	40 (38.1)
Unknown, n (%)	0 (0.0)	7 (0.5)	0 (0.0)	0 (0.0)
Lymph node yield				
<12, n (%)	287 (19.8)	323 (23.0)	36 (8.1)	13 (12.4)
≥12, n (%)	1166 (80.2)	1073 (76.5)	408 (91.9)	92 (87.6)
Unknown, n (%)	0 (0.0)	7 (0.5)	0 (0.0)	0 (0.0)
Mucinous (colloid) type	98 (6.7)	61 (4.4)	29 (6.5)	6 (5.7)
Lymphovascular invasion	123 (8.5)	83 (5.9)	56 (12.6)	6 (5.9)

Perineural invasion	30 (2.1)	29 (2.1)	83 (18.7)	7 (6.8)

Data are median [IQR], or n (%).

YNCH, Yunnan Cancer Hospital.

SYSU6, the Sixth Affiliated Hospital of Sun Yat-sen University.
