# Supplementary material for: Prediction models of colorectal cancer prognosis incorporating perioperative longitudinal serum tumor markers: a retrospective longitudinal cohort study
Source: BMC Med. 2023 Feb 21;21:63. doi: 10.1186/s12916-023-02773-2 (PMC9942392; doi:10.1186/s12916-023-02773-2)
Supplement: Supplementary file 2 — Additional file 2: Figure S1. [Kaplan-Meier curves of overall survival for the preoperative groups of CEA (a), CA19-9 (b), and CA125 (c) in colorectal cancer patients]. Figure S2. [Kaplan-Meier curves of overall survival for the first postoperative groups of CEA (a), CA1 -9 (b), and CA125 (c) in colorectal cancer patients]. Figure S3. [Longitudinal trajectories of CEA (a), CA19-9 (b) and CA125 (c) of survived and died patients]. Figure S4. [The eigenfunctions of FPCA for CEA (a), CA19-9 (b), and CA125 (c)]. Figure S5. [The first five eigenfunctions for CEA (a), CA19-9 (b), and CA125 (c) based on MFPCA of the three markers]. Figure S6. [Variable importance of preoperative CEA&CA19-9&CA125 model (a) and longitudinal CEA&CA19-9&CA125 model (b)]. Figure S7. [ROC curves of the preoperative, postoperative and longitudinal CEA&CA19-9&CA125 models at 60 months after surgery for internal validation (a) and external validation (b)]. Figure S8. [Personalized dynamic prediction for the survival probability of patient A (a), patient B (b) and patient C (c) based on longitudinal CEA&CA19-9&CA125 model]. Figure S9. [AUC (a) and BS (b) of the prediction models with MSI status at 24 to 60 months after surgery for external validation]. [file 12916_2023_2773_MOESM2_ESM.pdf]

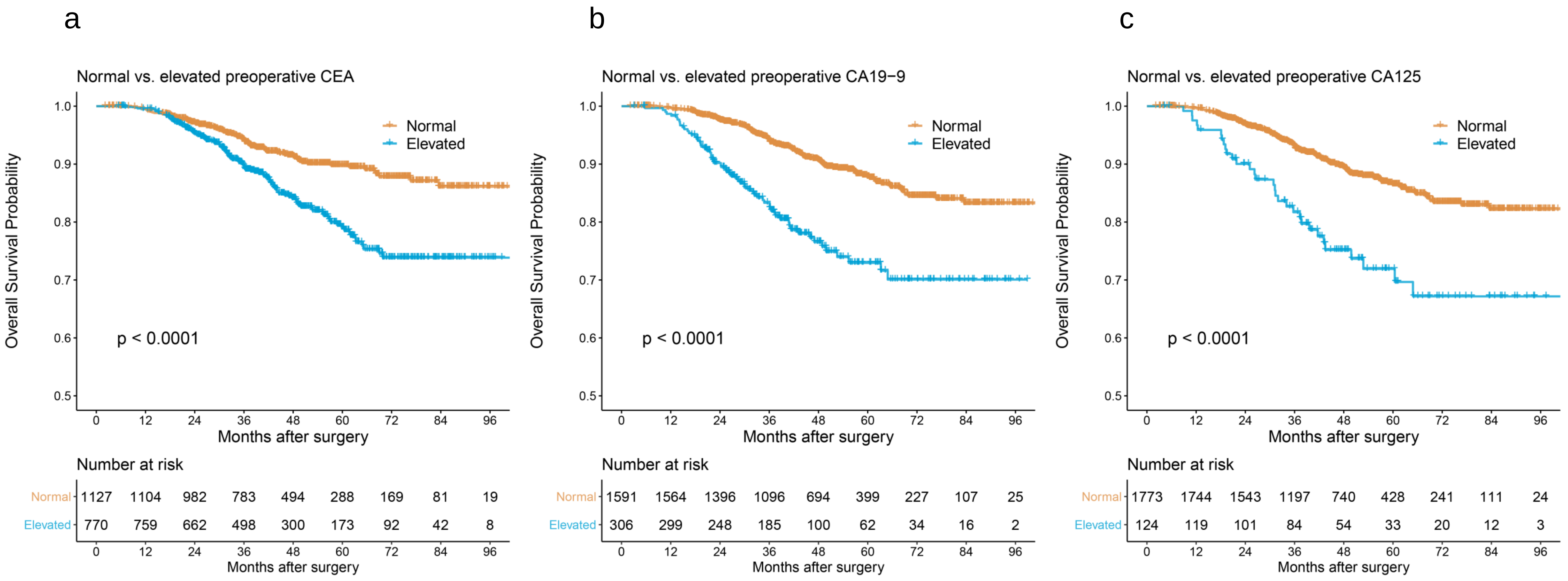

Figure S1. Kaplan-Meier curves of overall survival for the preoperative groups of CEA (a), CA19-9 (b), and CA125 (c) in colorectal cancer patients

P values were calculated using log-rank test

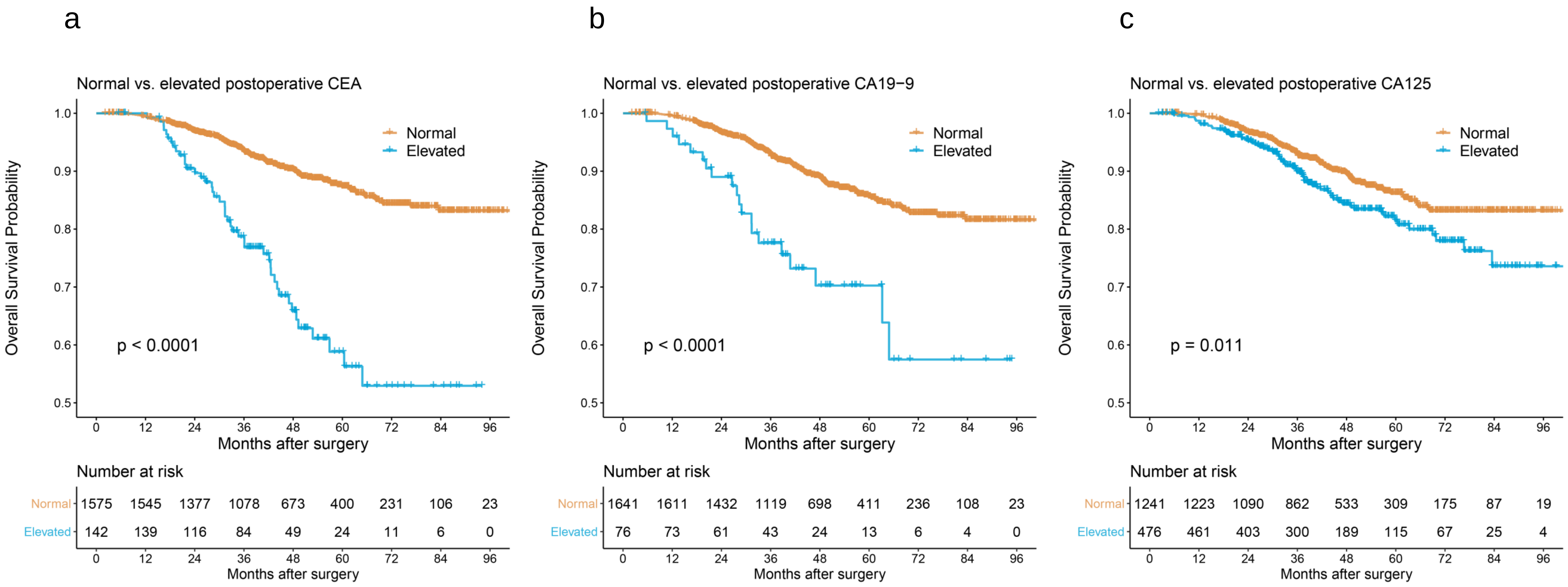

Figure S2. Kaplan-Meier curves of overall survival for the first postoperative groups of CEA (a), CA19-9 (b), and CA125 (c) in colorectal cancer patients

P values were calculated using log-rank test

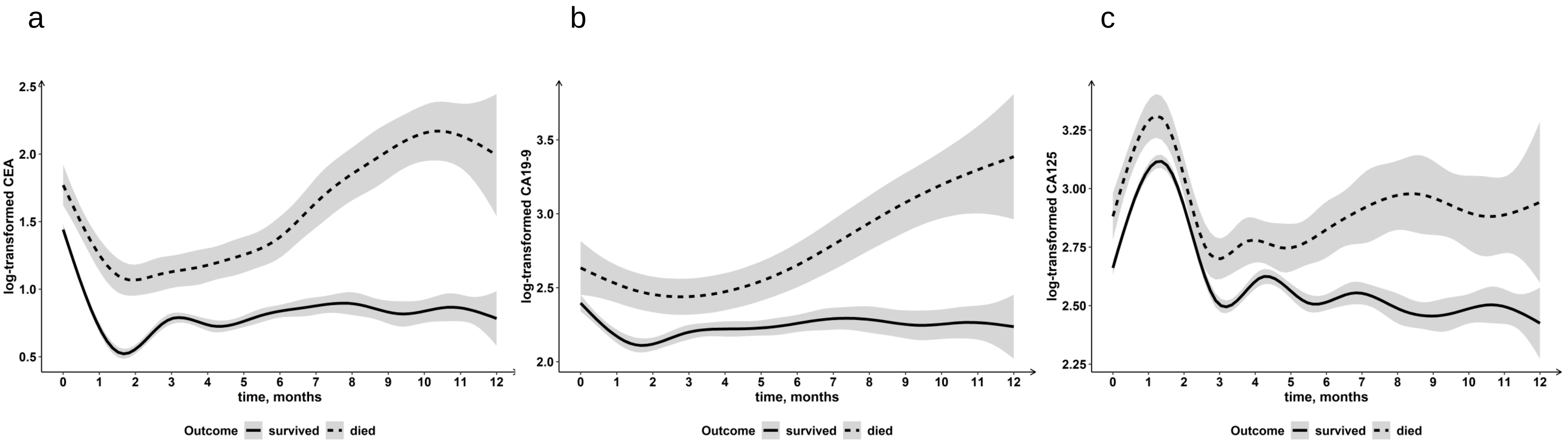

Figure S3. Longitudinal trajectories of CEA (a), CA19-9 (b) and CA125 (c) of survived and died patients

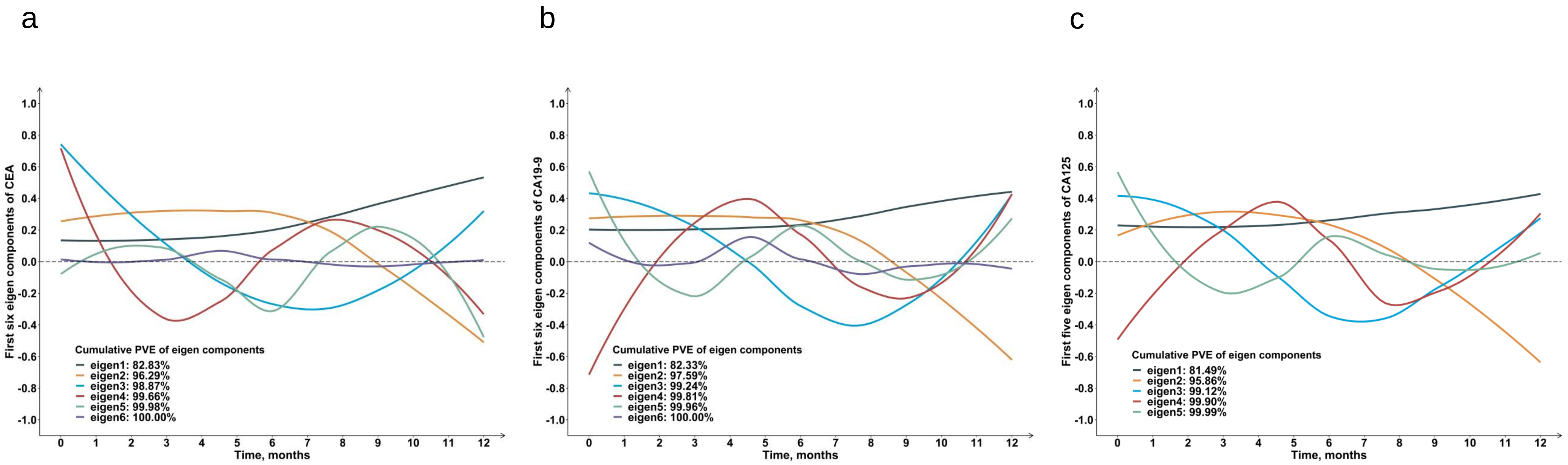

Figure S4. The eigenfunctions of FPCA for CEA (a), CA19-9 (b), and CA125 (c)
FPCA, functional principal component analysis; PVE, percentage of variance explained

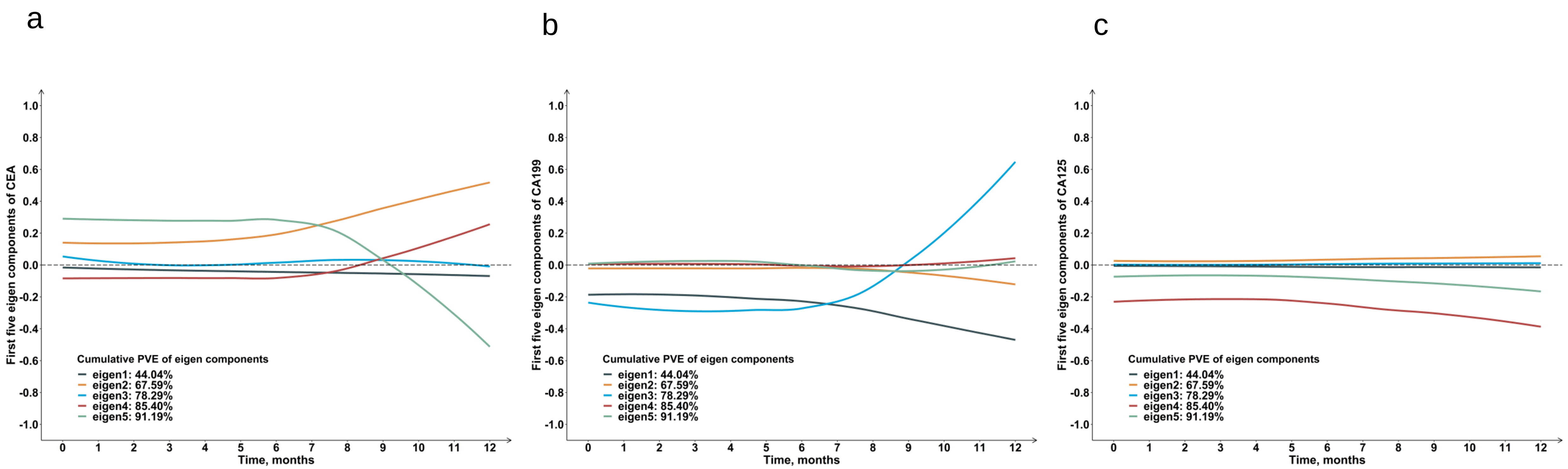

Figure S5. The first five eigenfunctions for CEA (a), CA19-9 (b), and CA125 (c) based on MFPCA of the three markers

MFPCA: multivariate principal components analysis; PVE, percentage of variance explained

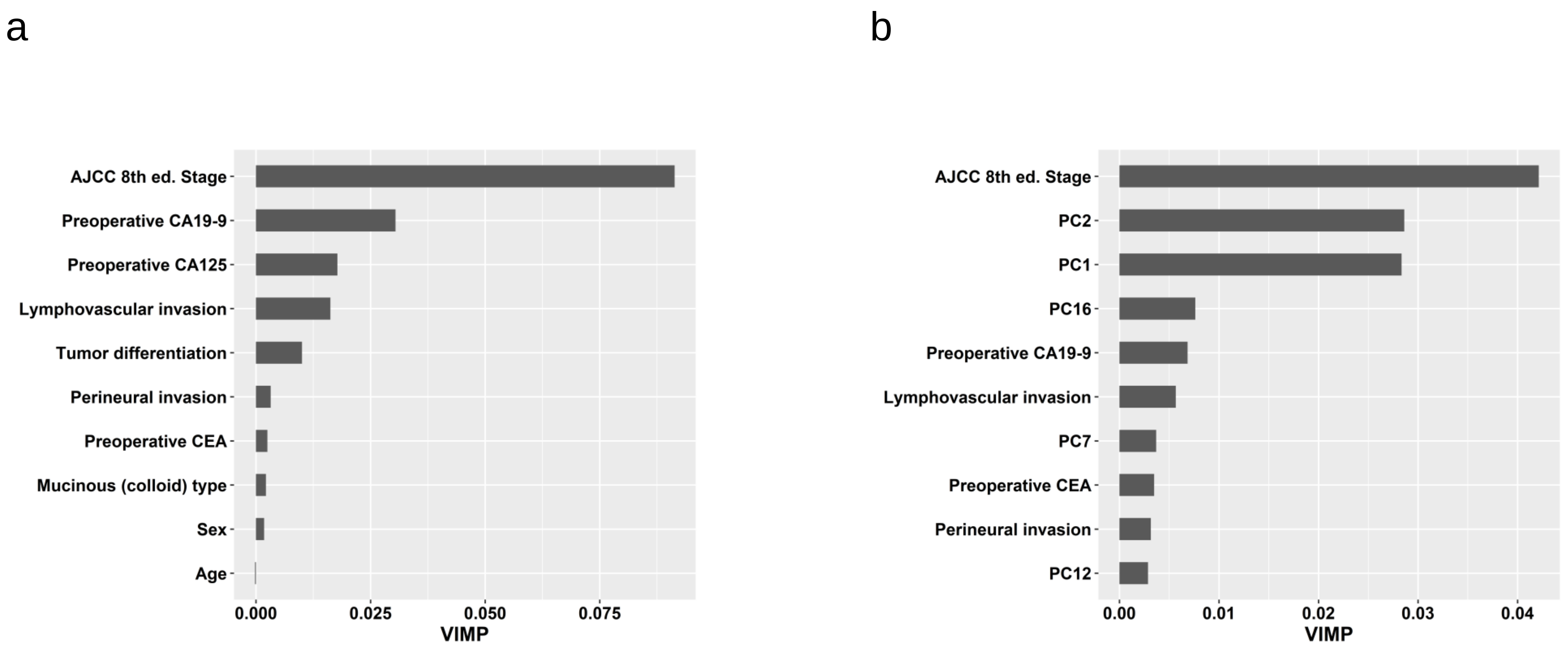

Figure S6. Variable importance of preoperative CEA&CA19-9&CA125 model (a) and longitudinal CEA&CA19-9&CA125 model (b)

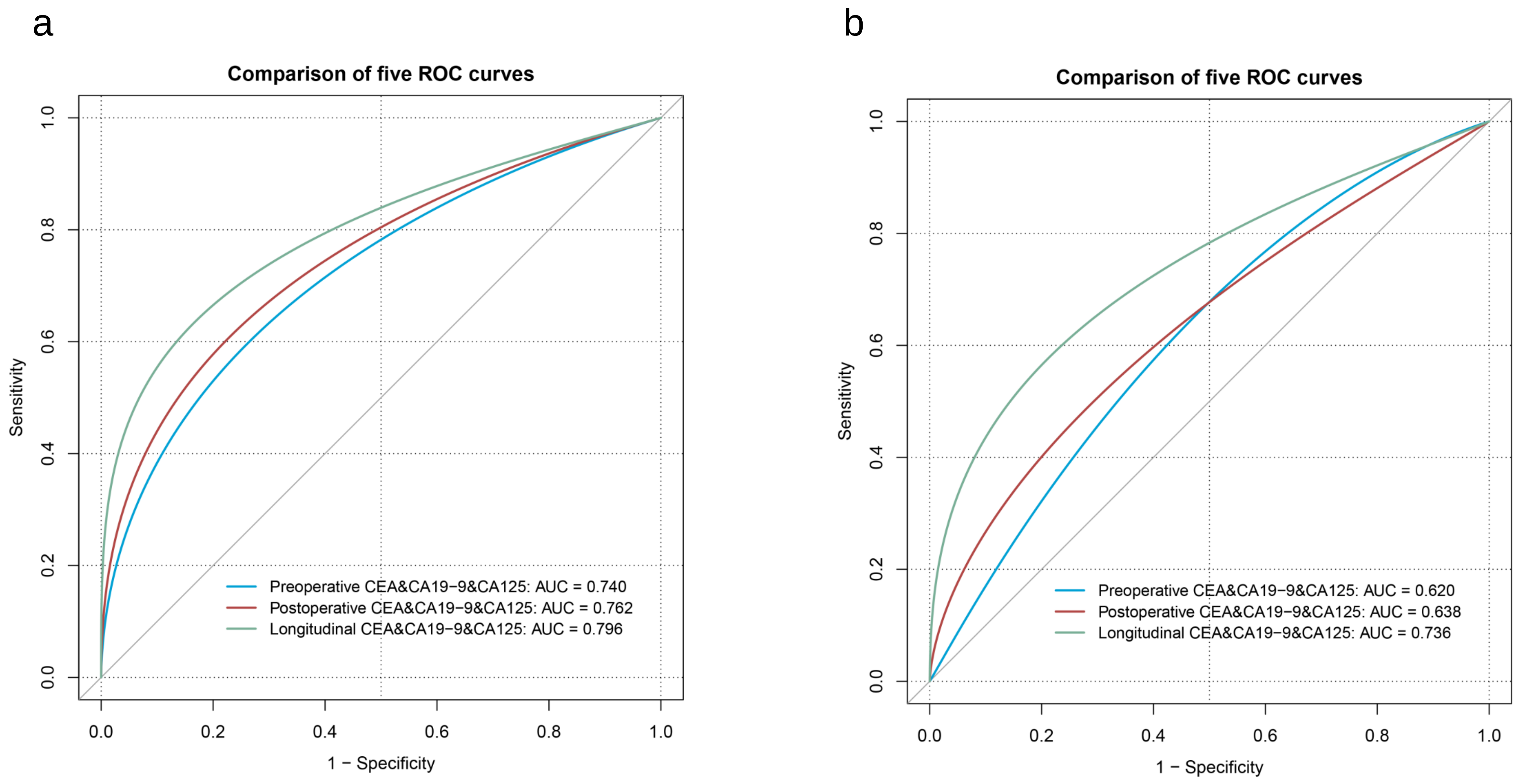

Figure S7. ROC curves of the preoperative, postoperative and longitudinal CEA&CA19-9&CA125 models at 60 months after surgery for internal validation (a) and external validation (b)
AUC, area under the receiver operating characteristic curve

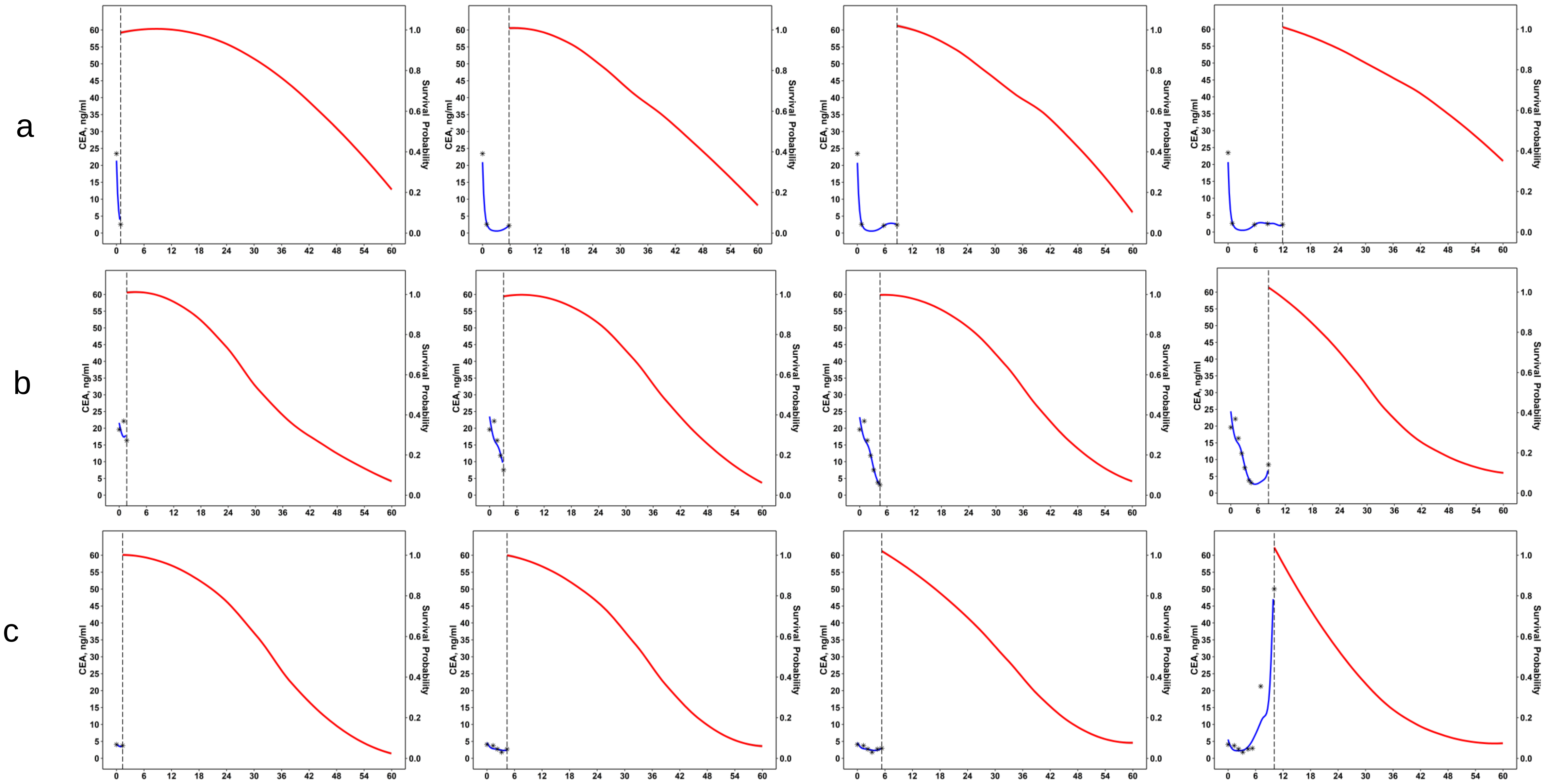

Figure S8. Personalized dynamic prediction for the survival probability of patient A (a), patient B (b) and patient C (c) based on longitudinal CEA&CA19-9&CA125 model
The time point of measurement is represented in the vertical dotted line, left of it is the observed (spot) and estimated (line) CEA, and right is the predicted survival probability

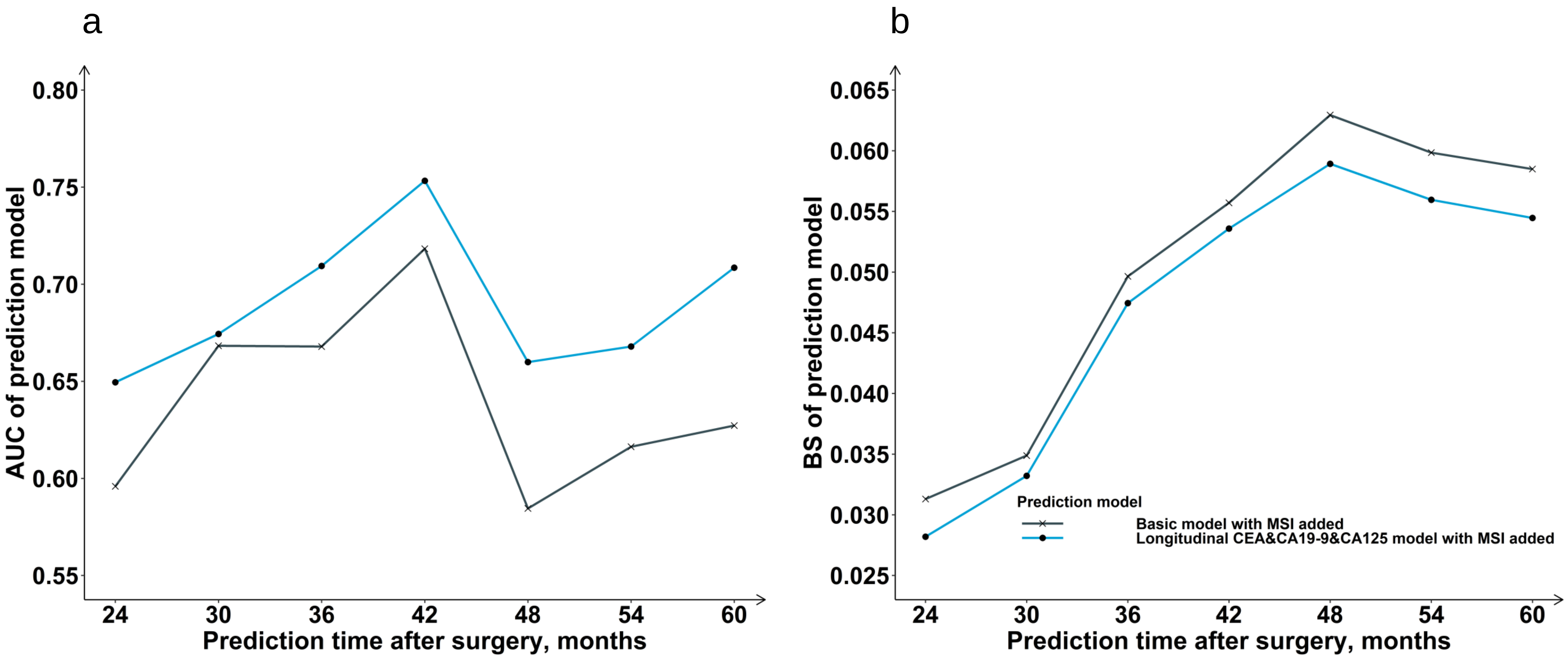

Figure S9. AUC (a) and BS (b) of the prediction models with MSI status at 24 to 60 months after surgery for external validation

AUC, area under the receiver operating characteristic curve; BS, brier score

The Basic model with MSI added had all variables in the basic model as well as MSI status.

The longitudinal CEA&CA19-9&CA125 model with MSI added had all variables in the longitudinal CEA&CA19-9&CA125 model as well as MSI status.
